# Social leisure time activities as a mediating link between self-reported psychological symptoms in adolescence and psychiatric morbidity by young adulthood: the Northern Finland 1986 Birth Cohort study

**DOI:** 10.1007/s00787-022-02107-2

**Published:** 2022-11-22

**Authors:** Johanna Timonen, Mika Niemelä, Helinä Hakko, Anni Alakokkare, Sami Räsänen

**Affiliations:** 1 Faculty of Medicine, Research Unit of Clinical Neuroscience, University of Oulu, Psychiatry, Finland; 2 Department of Psychiatry, Oulu University Hospital, Oulu, Finland; 3 Faculty of Medicine, Center for Life Course Health Research, University of Oulu, Oulu, Finland

**Keywords:** Adolescence, Social leisure time activity, Youth self-report, Mental health, Symptoms

## Abstract

Research indicates that adolescent psychological symptoms are associated with subsequent mental health disorders. Studies also show the association of leisure activity with improved current and future mental health. However, research is limited on whether social leisure time activity is a mediating link in the association between psychological symptoms and later psychiatric morbidity. We examined whether adolescence-related social leisure time activity, per se, is a mediating link in the association between adolescent psychological symptoms and later psychiatric morbidity. The study population was based on the Northern Finland Birth Cohort 1986 Study (NFBC 1986; *n* = 6709; 3227 males). Psychological symptoms at age 15–16 years were measured with the Youth Self Report (YSR) questionnaire. Study participants’ psychiatric morbidity by the age of 33 years was assessed using the diagnoses from the nationwide health care registers. Our results showed an association between psychological symptoms and leisure time activities that varied depending on the level of social activity. Leisure time activity was found to be a mediating link between psychological symptoms in adolescence and psychiatric disorders in early adulthood. Adolescence-related leisure time activities, which differed with regard to social interactions, appeared to serve as a mediating link between adolescent psychological symptoms and later onset of psychiatric disorders. Socially active leisure time during adolescence is related to better long-term mental health, while socially inactive leisure time associates with the likelihood of later psychiatric morbidity. To prevent psychiatric disorders, enhancing such leisure time activities in society is highly recommended.

**Supplementary Information:**

The online version contains supplementary material available at 10.1007/s00787-022-02107-2.

## Introduction

Psychological symptoms occurring during childhood and adolescence are shown to associate with later psychiatric morbidity*,* particularly in depression and anxiety disorders [1–3]. The processes of development of these symptoms into psychiatric disorders are related to several biological and environmental factors which may be either protective or risk-increasing [4] Biological factors, such as genetic vulnerability for psychiatric disorders, have been a continuous research interest for decades [5, 6]. The impact of different environmental factors on mental health disorders is increasingly emphasized in literature [7, 8].

From the family perspective, environmental factors can be categorized into family-related factors and factors outside of the family [9, 10]. Family-related demographics, such as socioeconomic status, parental educational level and marital status, are shown to impact on psychiatric morbidity of offspring [11, 12]. In addition, family functioning (i.e., communication, interactions and relationships within family) is associated with mental health among offspring [13, 14]. Environmental factors outside of the family include various social environments, such as school and neighborhood. These social environments have been shown to have a positive effect on the development of the young person but may also have unfavorable effects by exposing to adverse events, such as bullying or insecurity [1, 15].

Adolescence is a significant age stage in terms of psychological development and later mental health. During this a lot of changes are happening in the development and life of a young person. Maintaining balance during the changes of puberty is challenging. Social relationships and leisure time usually form a significant part of young people’s daily routines and, thus, have a significant impact on young people’s development and general well-being [16, 17] Leisure time activity has been reported to be an environmental factor associated with mental health of children and adolescents [18, 19]. Hobbies such as sports and arts generally associate with positive mental health of those involved [20, 21]. Children and adolescents with active leisure time activities have been documented to have fewer psychological symptoms than their peers without these activities [22–24]. People’s behavior and perception of themselves is widely linked to those social groups, where they belong. Social leisure time, therefore, has significant health effects and factors promoting well-being [25]

On the other hand, psychological symptoms of disturbing level have been found to reduce participation in leisure time activities and social relationships. For example, [26] reported that higher levels of internalizing symptoms predicted lower levels of organized activity participation among young people. They interpreted that especially depressive symptoms such as low energy, loss of interest and negative mood can result in less involvement in activities. In addition, [27] showed that children and adolescents with high levels of depressive symptoms may be less active but they may be more involved with increased screen time, which may in turn be associated with increased depressive symptoms.

Recently, we showed at the population level that socially active leisure time in adolescence (ages 15–16 years) was associated with lower incidence of diagnosed psychiatric disorders by young adulthood (from age 16–33 years) [28]. Our finding raised the further research question of whether social leisure time activity, per se*,* is a mediating link in the association between psychological symptoms and later psychiatric morbidity of the study participants. This study will add new perspective to development of psychological symptoms to diagnosed psychiatric disorders.

Thus, in the current study, following the segmentation approach of the mediation analysis [29], three research objectives and hypotheses were formed: (1) psychological symptoms in adolescence impact on the level of social leisure time activity (SLA), (2) the level of SLA in adolescence affects the onset of psychiatric disorders in young adulthood, and, consequently, (3) SLA in adolescence mediates the relationship between psychological symptoms in adolescence and the onset of psychiatric disorders at ages 16–33 years.

## Methods

### Study population

**Fig. 1:**
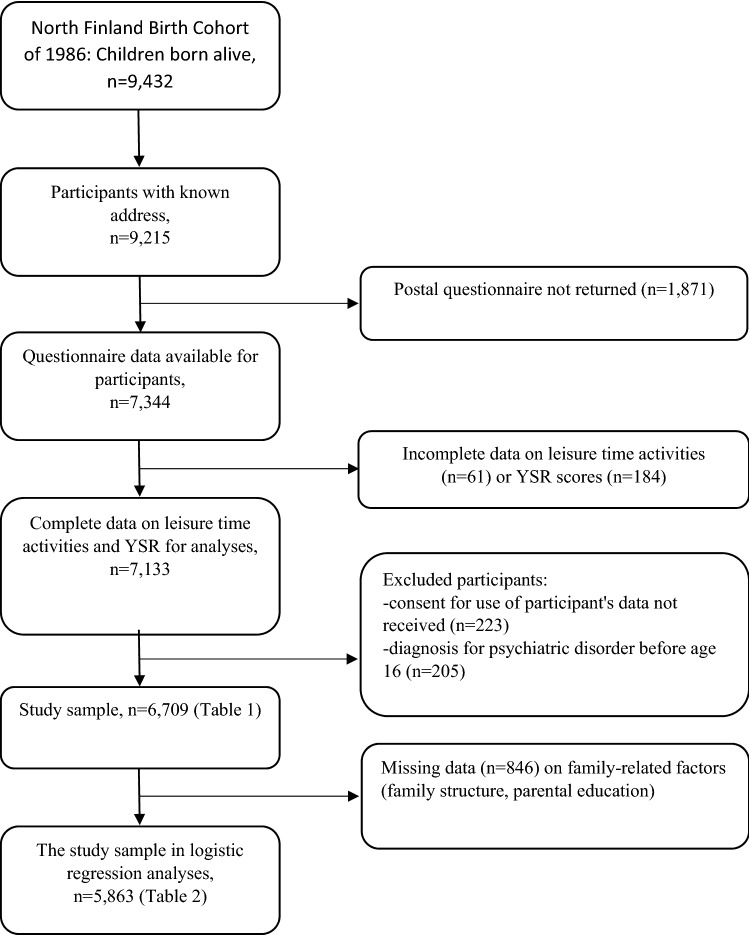
Sampling of the data

The study population consists of the members of the 1986 Northern Finland Birth Cohort Study (NFBC1986). The Northern Finland Birth Cohort 1986 is a longitudinal follow-up study. It covers all children born alive in Oulu and Lapland from 1 July 1985 to 30 June 1986, altogether 9432 children (4865 boys). For follow-up surveys, 9215 cohort members had a known mailing address in 2001 when the 16-year follow-up study was conducted (https://www.oulu.fi/nfbc/node/18149) Of them, 7344 participants participated in the postal survey at the age of 15–16. In that survey, questions related to Youth Self-Report (YSR) [30] and leisure time activities were answered by 7133 cohort members, of whom 6910 gave permission to use their data for research purposes. The life-time psychiatric disorders of the cohort members are based on the nationwide Care Register for Health Care data covering all inpatient treatment from birth until 2018 and all specialized level outpatient visits for the years 1998–2018. For the purpose of this study, 201 cohort members were excluded because of mental disorder diagnoses set before 16 years of age. Thus, as shown in Fig. 1, the current study comprised 6709 participants, covering 71% of the total 1986 Birth Cohort. Due to missing data on some family-related factors, 5863 participants were included in the logistic regression analyses.

### Data sources

The psychiatric disorders were based on the data of specialized level outpatient visits available for the years 1998–2018 and on the inpatient data from the Care Register for Health Care (CRHC) from birth of the cohort members until 2018. The main and secondary diagnoses were taken into account in the screening for psychiatric disorders of the cohort members.

Data on parental mental disorders (F00–F69, F80–F99, main and secondary diagnoses) were defined based on the information from the following national registers: inpatient treatment from the health care register (until 2018), specialized level outpatient visits (1998–2018) and primary health care from the Care Register for Health Care (2011–2018) and disability pension data from the Finnish Center for Pensions (until 2016).

Cohort study participants and their parents were asked for signed consent for use of the data for research purposes. The research was approved by the Ethical Evaluation Board of the Department of Health and Welfare (§28/2009), the Ethical Committee of the Northern Ostrobothnia Hospital District (EETTMK: 108/2017) and the Faculty of Medicine of the University of Oulu, Oulu, Finland. In addition, the administration of each registry gave permission to use individual-level registry-based information for scientific research.

## Measures

### The level of social leisure time activity in adolescence

The study participants were categorized into three hierarchical and mutually exclusive groups of social leisure activity (SLA) based on their answers to leisure activity questions in a postal questionnaire completed at 15–16 years of age. The following SLA groups were formed: 1. High level of social leisure activity (high SLA) (*n* = 2111), i.e., the participant belongs to a community or sports club (of the high SLA group, 1,653 (78.6%) belonged to a sports club and 593 (28.1%) belonged to a parish club or scouts and 135 (6.4%), belonged to a sports club and to a parish club or scouts), 2. Middle level of social leisure activity (middle SLA; *n* = 4485), i.e., the participant has leisure time activity, where other people have to be considered, but he/she does not belong to a community or a sports club, such as dancing or badminton, and 3. Low level of social leisure activity (low SLA) (*n* = 215), i.e., the participant only has activities that can be done alone, such as reading, cycling or listening to music. The detailed description of the definition of SLA groups is presented in our earlier publication [28].

### Psychological symptoms in adolescence according to Youth Self Report (YSR) questionnaire

The 16-year follow-up survey was conducted between the years 2001 and 2002 ((https://www.oulu.fi/sites/default/files/postikysely_eng_1.pdf)). It contained a self-assessment postal questionnaire on health and well-being, including questions on physical health, psychosocial well-being, living habits and the Youth Self Report (YSR) questionnaire.

The YSR is a self-report questionnaire for 11–18 years developed by [31]. In the 16-year postal survey, the items of the YSR were based on 1991 version. The categorization of the 88 items into eight subscales was based on the 2001 version of the modified scale [30, 32, 33]. The adolescent assesses on how each statement comes true now or came true in the last 6 months. The eight subscales of the YSR include scales for (1) anxious/depressed (13 items), (2) withdrawn/depressed (7 items), (3) somatic complaints (10 items), (4) social problems (10 items), (5) thought problems (12 items), (6) attention problems (7 items), (7) rule-breaking behavior (12 items) and (8) aggressive behavior (17 items).

Furthermore, three combined scales were formed by combining selected subscales, the first named as internalizing symptoms scale (including subscales for anxious/depressed, withdrawn/depressed and somatic complaints) and the second as externalizing symptoms scale (including subscales for rule-breaking behavior and aggressive behavior). The rest of the subscales (social problems, thought problems and attention problems) were included in the combined scale “other psychological symptoms”. The YSR total score included the scores of all eight subscales.

For statistical analyses, the sum score of the total YSR, of each subscale and of each combined scale was calculated. If less than three items were missing in a subscale, missing values were replaced by the mean of the subscale from that study participant. If three or more items were missing, the corresponding subscale was removed for that person. Mean-imputated subscales were used to compute YSR total score as well as internalizing, externalizing and other symptoms.

For further statistical analyses, the sum scores of the YSR scales were dichotomized at 82nd percentile of the distribution of a scale [34]. In that study*,* the 82nd percentile was suggested to be the optimal cutoff score for indicating the presence of a mental health condition. In our study, these cutoffs were calculated separately for males (total score 33.0, internalizing symptoms 10.5, externalizing symptoms 14.0 and other symptoms 10.0) and females (total score 47.0, internalizing symptoms 18.0, externalizing symptoms 16.0 and other symptoms 14.1). The gender-specific values were calculated, because it is known that psychological symptoms are linked to age [35–37].

### Psychiatric disorders of the study participants

The main outcome variables were diagnosed psychiatric disorders of the study subjects set for the first time at ages 16–33 years. In addition to any psychiatric disorders (ICD-10 codes: F00–F99), the following main diagnostic categories were analyzed: mental and behavioral disorders due to psychoactive substance use (F10–F19); schizophrenia, schizotypal, delusional and other non-mood psychotic disorders (F20–F29); mood (affective) disorders (F30–F39); anxiety, dissociative, stress-related, somatoform and other nonpsychotic mental disorders (F40–F49), and behavioral and emotional disorders with onset usually occurring in childhood and adolescence (F90–F98). These psychiatric diagnostic categories were chosen, because the preliminary analyses (data not presented) showed them to be the most prevalent diagnostic categories, justifying the purpose of the subsequent statistical analysis.

### Family-related covariates

In our study, the selection of the family-related covariates as indicators for environmental factors was based on the previous literature reporting significant findings on their association with mental health and welfare of offspring; single parenthood [38], parents’ education [39] and parental mental health problems [11]. Information of family structure and parents’ education was based on a postal questionnaire that the parents filled in when the cohort participants were aged 15–16 (https://www.oulu.fi/sites/default/files/postikysely_eng_2.pdf). In the current study, the variable biological parents not living together means that the parents have never lived together, they are divorced from marriage or from co-habiting, or a parent has died. Parental educational level indicates that at least secondary level of education was completed by the study participant’s mother or father. The data of parent’s mental disorders (F00–F69, F80–F99, main and secondary diagnoses) were extracted from the following nationwide registers: inpatient treatment from the Care Register for Health Care (until 2018), specialized level outpatient visits (1998–2018) and primary care (2011–2018), from the Care Register for Health Care and information of disability pensions from the Finnish Center for Pensions (until 2016). The distribution of the family-related covariates is presented in supplementary material. See Appendix 1.

### Statistical methods

**Fig. 2:**
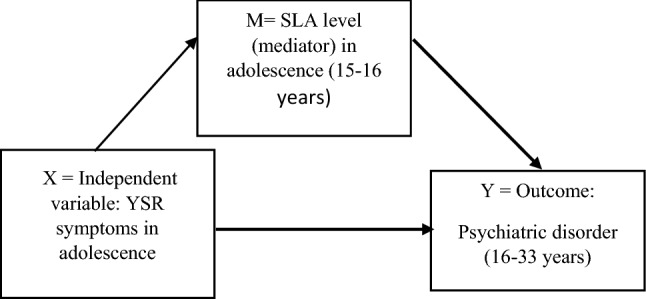
Segmentation approach of the mediaton analysis (Memon et al. 2018) applied in our study

In our study, the analyses relating to the concept of mediator was based on the segmentation approach described by Memon et al. [29] 2018. In the segmentation approach (Fig. 2), three research aims, and hypotheses are developed as follows: (1) independent variable (X, i.e., in our study, psychological symptoms in adolescence) effects mediator (M, i.e., social leisure time activity in adolescence), (2) mediator (M) effects outcome variable (Y, i.e., psychiatric morbidity by young adulthood) and (3) M mediates the relationship between X and Y. Statistical significance of group differences in categorical variables was assessed with Pearson Chi-square test and in continuous variables with Mann–Whitney *U* test. The association of the level of social leisure time activity and psychological symptoms (YSR scores in adolescence) with psychiatric disorder diagnosed at ages 16 through 33 years was examined using a binary logistic regression analysis after controlling for gender and family-related covariates (biological parents living together, parental education level and parental psychiatric disorders) of the study participants).

The primary analyses relating to the YSR scale were based on the cutoff point of 82nd percentile of distribution, which is suggested to indicate the presence of a mental health condition [34]. This cutoff point was applied to the sum score of the total YSR scale and to sum scores of the subscales for internalizing symptoms, externalizing symptoms, and other symptoms. In logistic regression analyses, Model 1 was formed for the total YSR scale and Model 2 for its three subscales (internalizing, externalizing and other symptoms). To evaluate the robustness of our findings, we also conducted sensitivity analyses, in which YSR sum scores were entered to the models as continuous variables (see Supplemental Table 1).

In terms of the eight subscales of the YSR (anxious/depressed, withdrawn/depressed, somatic complaints, social problems, thought problems, attention problems, rule-breaking behavior, aggressive behavior (17 items), the association of these subscales with the levels of SLA and psychiatric disorder diagnosed between ages 16–33 years were explored and visualized with boxplots (see Supplemental Figures 1-2). In additional analysis (see Supplemental Table 2), the association of each of the eight subscales (using 82nd cutoff for sum score) and the level of SLA in adolescence to likelihood of later psychiatric disorder was examined after controlling for covariates.

The statistical software used in analyses was IBM SPSS Statistics 25.

## Results

### A*ssociation of psychological symptoms with social leisure time activity in adolescence*

In the current study sample (*n* = 6709), the proportion of study participants exceeding the cutoff of 82nd percentile (indicating the presence of a mental health condition) of the total YSR scores was 26.2% (*n* = 54/206) in low-SLA, 18.5% (*n* = 817/4422) in middle-SLA and 15.1% (*n* = 315/2081) in high-SLA groups (*p* <  = 0.001). The corresponding proportions for internalizing psychological symptoms were 33.0% (*n* = 31), 19.0% (*n* = 848) and 15.7% (*n* = 330) (*p* < 0.001) and for externalizing symptoms, 14.6% (*n* = 31), 21.1% (*n* = 943), and 16.4% (*n* = 343) (*p* < 0.001). In psychological symptoms other than internalizing or externalizing symptoms, the proportions were 32.5% (*n* = 67) in low-SLA, 19.5% (*n* = 865) in middle-SLA, and 16.4% (*n* = 342) in high-SLA groups of the study participants (*p* < 0.001).

### SLA and psychological symptoms in adolescence in relation to later psychiatric morbidity

Table 1 shows the incidence of psychiatric disorders diagnosed at ages 16–33 years of the study participants (*n* = 6709) in relation to the level of SLA and psychological symptoms assessed in adolescence (ages 15–16). Overall, the incidence of any psychiatric disorders diagnosed for the first time at 16–33 years of age was 14.6% (*n* = 979), the incidence (from highest to lowest) being 7.7% (*n* = 525) in anxiety, 7.6% (*n* = 511) in affective disorders, 2.9% (*n* = 197) in substance use, 1.4% (*n* = 96) in psychotic and 1.1% (*n* = 76) in behavioral disorders.

**Table 1:** Levels of social leisure time activity (SLA) and self-reported psychological symptoms in adolescence (ages 15–16 years) in relation to incidence psychiatric disorders by young adulthood (ages 16–33 years), the 1986 Northern Finland Birth Cohort Study (*n* = 6709)

	F00-F99: Any psychiatric disorder			F10-F19: Substance use disorders			F20-F29: Psychotic disorders		
	Yes (*n* = 979)	No (*n* = 5730)		Yes (*n* = 197)	No (*n* = 6512)		Yes (*n* = 96)	No (*n* = 6613)	
	*n* (%)	*n* (%)	*p*-value	*n* (%)	*n* (%)	*p*-value	*n* (%)	*n* (%)	*p*-value
Social leisure activity^1^			< 0.001			0.017			0.611
Low-SLA	45 (21.8)	161 (78.2)		6 (2.9)	200 (97.1)		4 (1.9)	202 (98.1)	
Middle-SLA	689 (15.6)	3733 (84.4)		148 (3.3)	4274 (96.7)		66 (1.5)	4356 (98.5)	
High-SLA	245 (11.8)	1836 (88.2)		43 (2.1)	2038 (97.9)		26 (1.2)	2055 (98.8)	
YSR^2^ total									
≥ 82nd pctl			< 0.001			< 0.001			< 0.001
Yes	280 (23.6)	906 (76.4)		68 (5.7)	1118 (94.3)		34 (2.9)	1152 (97.1)	
No	699 (12.7)	4824 (87.3)		129 (2.3)	5394 (97.7)		62 (1.1)	5461 (98.9)	
YSR^2^ internalizing problems									
Med (IQR)	10.0 (5.0–16.0)	7.0 (4.0–12.0)	< 0.001	9.0 (5.0–16.0)	8.0 (4.0–13.0)	< 0.001	10.0 (5.5–17.0)	8.0 (4.0–13.0)	< 0.001
≥ 82nd pctl			< 0.001			< 0.001			< 0.001
Yes	273 (22.1)	963 (77.9)		56 (4.5)	1180 (95.5)		34 (2.8)	1202 (97.2)	
YSR^2^ externalizing problems									
≥ 82nd pctl			< 0.001			< 0.001			< 0.001
Yes	266 (20.4)	1035 (79.6)		75 (5.8)	1226 (94.2)		33 (2.5)	1268 (97.5)	
YSR^2^ other problems									
≥ 82nd pctl			< 0.001			< 0.001			< 0.001
Yes	300 (23.6)	969 (76.4)		81 (6.4)	1188 (93.6)		39 (3.1)	1230 (96.9)	

	F30-F39: Affective disorders			F40-F49: Anxiety			F90-F98: Behavioural disorders		
	Yes (*n* = 511)	No (*n* = 6198)		Yes (*n* = 525)	No (*n* = 6184)		Yes (*n* = 76)	No (*n* = 6633)	
	*n* (%)	*n* (%)	*p*-value	*n* (%)	*n* (%)	*p*-value	*n* (%)	*n* (%)	*p*-value
Social leisure activity^1^			< 0.001			< 0.001			0.007
Low-SLA	22 (10.7)	184 (89.3)		31 (15.0)	175 (85.0)		7 (3.4)	199 (96.6)	
Middle-SLA	372 (8.4)	4050 (91.6)		368 (8.3)	4054 (91.7)		49 (1.1)	4373 (98.9)	
High-SLA	117 (5.6)	1964 (94.4)		126 (6.1)	1955 (93.9)		20 (1.0)	2061 (99.0)	
YSR^2^ total									
≥ 82nd pctl			< 0.001			< 0.001			< 0.001
Yes	164 (13.8)	1022 (86.2)		143 (12.1)	1043 (87.9)		31 (2.6)	1155 (97.4)	
No	347 (6.3)	5176 (93.7)		382 (6.9)	5141 (93.1)		45 (0.8)	5478 (99.2)	
YSR^2^ internalizing problems									
Med (IQR)	11.5 (6.0–18.0)	7.0 (4.0–12.0)	< 0.001	11.0 (6.0–17.0)	7.2 (4.0–12.0)	< 0.001	11.0 (5.5–17.0)	8.0 (4.0–13.0)	< 0.001
≥ 82nd pctl			< 0.001			< 0.001			< 0.001
Yes	166 (13.4)	1070 (86.6)		156 (12.6)	1080 (87.4)		26 (2.1)	1210 (97.9)	
YSR^2^ externalizing problems									
≥ 82nd pctl			< 0.001			< 0.001			< 0.001
Yes	153 (11.8)	1148 (88.2)		136 (10.5)	1165 (89.5)		34 (2.6)	1267 (97.4)	
YSR^2^ other problems									
≥ 82nd pctl			< 0.001			< 0.001			< 0.001
Yes	175 (13.8)	1094 (86.2)		151 (11.9)	1118 (88.1)		30 (2.4)	1239 (97.6)	

*Med* median, *IQR* interquartile range, *pctl* percentile

Participant can have diagnoses from several diagnosis groups. Levels of social activity are mutually exclusive

^1^SLA social leisure activity

^2^YSR Youth self-report

Yes, indicates the presence on psychiatric disorder

The level of SLA in adolescence was statistically significantly associated with the incidence of any and specific psychiatric disorders, except in psychotic disorders. The incidence of psychiatric disorders was highest in the low SLA group, being 21.8% in any psychiatric disorder (range from 1.9% in psychotic disorders to 15.0% in anxiety disorders). Furthermore, the incidence was lowest in the high SLA group, being 11.8% in any psychiatric disorder (range from 1.0% in behavioral disorders to 6.1% in anxiety disorders).

The proportion of study participants presenting clinical-level psychological symptoms in adolescence (the YSR scores ≥ 82nd percentile of the YSR scores) was statistically significantly associated with the incidence of any and all specific psychiatric disorder groups. In total YSR scores, the prevalence of study participants exceeding the 82nd percentile of the scores was 23.6% in any psychiatric disorders (range from 2.6% in behavioral disorders to 13.8% in affective disorders). The corresponding prevalence for internalizing symptoms was 22.1% in any psychiatric disorders (range from 2.1% in behavioral disorders to 13.4% in affective disorders), for externalizing symptoms 20.4% in any psychiatric disorder (range from 2.5% in psychotic disorders to 11.8% in affective disorders) and for other psychological symptoms, 23.6% in any psychiatric disorders (range from 2.6% in behavioral disorders to 13.8% in affective disorders).

### SLA and psychological symptoms in adolescence as predictors for later psychiatric morbidity

Table 2 presents the results of the adjusted logistic regression analyses examining the association of the level of social leisure time activity and self-reported psychological symptoms in adolescence (ages 15–16 years) with likelihood of psychiatric disorder diagnosed for the first time between ages 16 and 33 years. In Model 1, high total YSR scores in adolescence were related to increased likelihood for any or all specific psychiatric disorders among the study participants. Furthermore, low-SLA was associated with increased likelihood for any psychiatric disorders and for anxiety and behavioral disorders, while high-SLA was related to decreased likelihood for any psychiatric disorders, substance use and affective disorders. As Model 2 shows, in high-SLA group the likelihood for any psychiatric disorder and for affective disorders was decreased, whereas it was increased in those study participants with high scores in internalizing and other symptoms. High-SLA was associated with decreased likelihood for substance use disorders, while the likelihood for that disorder was increased in those with high scores in externalizing and other symptoms in adolescence. Increased likelihood for anxiety disorders was linked with low-SLA as well as with high scores in internalizing and other symptoms in adolescence. Furthermore, behavioral disorders associated with low-SLA and with high scores in externalizing symptoms. The increased likelihood for psychotic disorder was related to high scores of other psychological symptoms (social problems, thought problems and attention problems), besides than externalizing and internalizing, but not with any level of SLA in adolescence.

**Table 2:** Association of leisure time activity and self-reported mental symptoms in adolescence (defined as 82nd percentile of the YSR score) with likelihood of psychiatric disorder diagnosed by young adulthood, the 1986 Northern Finland Birth Cohort Study (*n* = 5863)

	F00-F99: Any psychiatric disorder	F10-F19: Substance use disorders	F20-F29: Psychotic disorders	F30-F39: Affective disorders	F40-F49: Anxiety	F90-F98: Behavioural disorders
	Adj OR (95% CI)	Adj OR (95% CI)	Adj OR (95% CI)	Adj OR (95% CI)	Adj OR (95% CI)	Adj OR (95% CI)
Model 1						
Social leisure activity^1^						
Low-SLA^2^	1.48 (1.02–2.15)	0.89 (0.38–2.06)	1.26 (0.45–3.55)	1.26 (0.77–2.06)	2.03 (1.33–3.12)	3.45 *(1.51–7.91)*
High-SLA^2^	0.80 (0.68–0.95)	0.55 (0.37–0.81)	0.83 (0.51–1.35)	0.74 (0.59–0.94)	0.82 (0.65–1.03)	1.15 (0.66–2.01)
YSR^3^ total > 82th percentile	2.01 (1.69–2.39)	2.26 (1.62–3.15)	2.65 (1.69–4.16)	2.37 (1.91–2.95)	1.72 (1.38–2.16)	2.80 *(1.69–4.63)*
Model 2						
Social leisure activity^1^						
Low-SLA^2^	1.42 (0.98–2.07)	0.90 (0.38–2.09)	1.19 (0.42–3.38)	1.18 (0.72–1.94)	1.92 (1.25–2.95)	3.92 (1.70–9.08)
High-SLA^2^	0.81 (0.68–0.96)	0.56 (0.38–0.83)	0.85 (0.52–1.39)	0.75 (0.60–0.95)	0.83 (0.66–1.04)	1.21 (0.69–2.12)
YSR^3^ internalizing symptoms ≥ 82nd percentile	1.25 (1.02–1.54)	0.91 (0.60–1.38)	1.52 (0.88–2.64)	1.44 (1.11–1.87)	1.45 (1.11–1.89)	1.19 (0.63–2.22)
YSR^3^ externalizing symptoms ≥ 82nd percentile	1.13 (0.93–1.37)	1.53 (1.05–2.22)	1.28 (0.76–2.17)	1.11 (0.86–1.43)	0.99 (0.76–1.29)	2.76 (1.57–4.85)
YSR^3^ other psychological symptoms ≥ 82nd percentile	1.77 (1.43–2.18)	2.39 (1.59–3.60)	2.14 (1.21–3.80)	2.00 (1.52–2.63)	1.46 (1.10–1.93)	1.39 (0.73–2.66)

ORs with 95% Confidence Intervals (CI) are calculated with logistic regression after adjusting for gender, family structure, and parental education when cohort members were 15–16 years of age, and the presence of parental psychiatric disorders until the end of 2018. In the models the level of social leisure activity (SLA) and high scores (≥ 82nd percentile) of the YSR scale/subscales in adolescence is associated with first onset any psychiatric and specific psychiatric disorders diagnosed between 16 and 33 years age. Participants can have psychiatric diagnoses from several diagnosis groups. Levels of social leisure activity are mutually exclusive

^1^reference category = Middle social leisure activity

^2^SLA social leisure activity

^3^YSR Youth self-report, high scores (≥ 82nd percentile) of the YSR score was defined to indicate the clinical cutoff point for the presence of a mental health condition

**Fig. 3:**
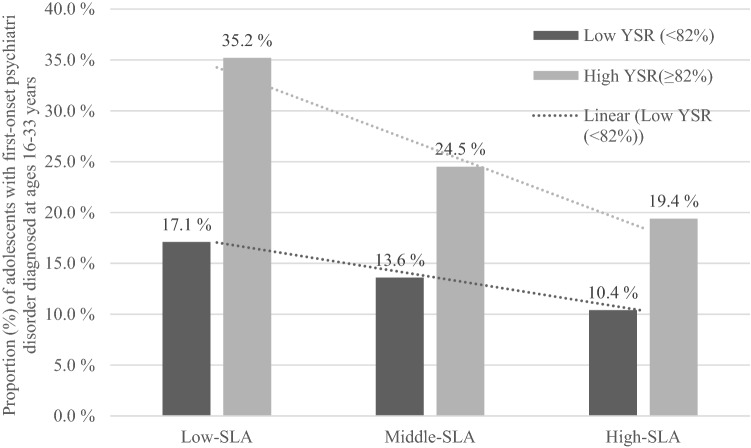
Proportion (%) of study participants with first onset of psychiatric disorder diagnosed at ages 16–33 years, by the level of social leisure activity (SLA) and the presence of psychological symptoms (total YSR scores; high ≥ 82nd percentile, low < 82nd percentile) in adolescence

Figure 3 illustrates the association of SLA and YSR in adolescence with the incidence of psychiatric disorder. Among the study participants with high YSR scores in adolescence, the highest incidence of any psychiatric disorder was observed in the low SLA group, 35.2%, degreasing to 19.4% in the high SLA group. Furthermore, among the study participants with a low YSR in adolescence, the incidence of psychiatric disorders was notably lower, being 17.1% in the low SLA group and dropping to 10.4% in the high SLA group.

A sensitivity analysis was conducted using continuous YSR scores instead of using the YSR 82nd percentile cutoffs in logistic regression models 1 and 2 (see Supplemental Table 1). There were no major changes in statistically significant associations of the levels of SLA and YSR scores with psychiatric disorders. The associations with YSR subscale for other psychological symptoms remained beyond statistical significance only in psychotic disorders and anxiety disorders.

## Discussion

Earlier research has provided wide evidence that active leisure time associates with positive mental health. There is also some research suggesting that leisure time activity can serve as protecting factor for psychiatric morbidity. However, the role of different types of hobbies in the development process of psychological symptoms into mental disorders is not clear. Identification of whether different types of leisure time activities mediate this transition would facilitate the development of preventive psychosocial actions and, thus, might reduce the need for later psychiatric treatment.

In this study we were able to investigate at the population level the role of leisure time as a link between psychological symptoms and psychiatric disorders. Regarding the mediation approach applied in our study, we were able to show the association between adolescence-related psychological symptoms and leisure time activities that differed in terms of the level of social activity. Among the study participants, a high level of psychological symptoms was associated with participation in socially low-level leisure time activity in adolescence. One indicator of this link was the observed association of high scores in internalizing symptoms with a low level of social leisure time activity (low-SLA). Respectively, a decreased level of internalizing symptoms was associated with a high level of social leisure time activity (high-SLA). These findings are consistent with previous studies reporting that individual’s depressive symptoms associate with spending less time in social interactions [40, 41].

Second, our study indicated the association between the level of social leisure time activity in adolescence and subsequent psychiatric disorders in early adulthood. The level of adolescence-related social leisure time activity was statistically significantly associated with the incidence of psychiatric disorders later in life and, specifically, with all analyzed psychiatric diagnostic categories, except in psychotic disorders. Our finding is in line with the studies documenting that participation in meaningful social leisure time activities has a positive longitudinal impact on mental health [3, 42]

Thirdly, adolescence-related leisure time activity as a mediating link between psychological symptoms in youth and psychiatric disorders in early adulthood was established in our study. This was seen, for example, in our finding that high total YSR score, together with low social leisure time activity, in adolescence was related to increased likelihood for any psychiatric diagnoses. Similarly, high social leisure time activity was related to decreased likelihood for psychiatric disorders regardless of high YSR scores in adolescence. We were able to adjust our analyses with commonly known family-related risk factors for psychiatric morbidity of the offspring, such as parents’ mental health disorders [12], parental educational level [39] and living with a single parent [38]. Thus, the impact of these risk factors on the occurrence of psychological symptoms as well as on the possibility to participate in leisure time activities in adolescence is controlled with regard to the onset of psychiatric disorders of the study subjects. Worth to noticing is, however, that when interpreting the findings of our study it is important to consider that severity of primary psychological symptoms may have affected to participation in hobbies and, thus, also be the main reason to manifestation of psychiatric disorders.

In general, previous research has widely acknowledged the association of adolescence-related psychological symptoms with later psychiatric morbidity [43–45]. For example, in the longitudinal study of Ormel and workgroup [46], it was found that adult mental health disorders are often preceded by psychological symptoms with onset during adolescence. The results of our study further suggest that the development of symptoms into diagnosed psychiatric disorders is mediated by a variety of factors, such as social leisure time activity in our study.

Exploring mediating links between symptoms and disorders gives new potential to construct targeted preventive actions for children and adolescents. In these actions, development of interaction skills and community’s ability to enhance individual’s sense of belonging to the community are primary targets, because they are known to associate with positive psychological outcomes and resilience among young people [47, 48]. For example, in the cohort study of Scardera and coworkers, 18 years with anxiety, depression or suicide risk reported fewer mental health problems after a year after receiving community support [49]. In addition, participation in organized leisure activities has shown to predict a low level of internalizing symptoms and to support positive mental health [50]. Early community-based activities have also been reported to have a long-term positive impact on mental health. Furthermore, participating in scouting in adolescence has been found to associate with better mental health at the age of 50 [51]. Their study reported that community-based support had a positive effect especially on children growing up in socially disadvantaged households. Therefore, it is highly important to organize various community leisure time activities especially for children and young people who are exposed to different adversities.

### Strengths and limitations

The strength of this study is the well-documented sample of young people who were followed for psychiatric morbidity from the age to 15–16 years until the age of 33 years. The 1986 Northern Finland Birth Cohort Study population can be considered as representative of Finnish adolescents. The national health care registers used in the current study have shown to be reliable for research purposes. [33]. Another strength of this study was that psychological symptoms were assessed with the Youth Self Report (YSR) questionnaire, which is a widely and commonly used measure for assessing youth emotional and behavioral problems. [52]. The third strength of this study is that mental disorder diagnoses of the study participants were extracted from nationwide healthcare registers including all inpatient hospitalizations and specialized level outpatient visits. The diagnoses in health care registers are always set by doctors and the diagnoses are based on criteria of the ICD classification of diseases.

A limitation of the study is that the small number of cases in the subgroup analyses may have caused lack of power in statistical analyses (Type II error). In addition, due to the many statistical comparisons performed in this study the possibility of chance findings (Type I error) cannot be excluded.

## Conclusion

Our study presents new information that adolescence-related leisure time activities that differ with regard to social interactions can serve as a mediating link between adolescent psychological symptoms and later onset of psychiatric disorders. Socially active leisure time during adolescence is related to better long-term mental health, while socially inactive leisure time associates with the likelihood of later psychiatric morbidity. To prevent psychiatric disorders, it is highly recommended that such leisure time activities are enhanced in society.

### Supplementary Information

Below is the link to the electronic supplementary material.

Supplementary file1 (DOCX 19 KB)

Supplementary file2 (DOCX 16 KB)

Supplementary file3 (DOCX 19 KB)

Supplementary file4 (DOCX 145 KB)

Supplementary file5 (DOCX 130 KB)
