# Correction to: Social leisure time activities as a mediating link between self-reported psychological symptoms in adolescence and psychiatric morbidity by young adulthood: the Northern Finland 1986 Birth Cohort study

**DOI:** 10.1007/s00787-023-02202-y

**Published:** 2023-04-12

**Authors:** Johanna Timonen, Mika Niemelä, Helinä Hakko, Anni Alakokkare, Sami Räsänen

**Affiliations:** 1 Faculty of Medicine, Research Unit of Clinical Neuroscience, University of Oulu, Psychiatry, Finland; 2 Department of Psychiatry, Oulu University Hospital, Oulu, Finland; 3 Faculty of Medicine, Center for Life Course Health Research, University of Oulu, Oulu, Finland

**Correction to: European Child & Adolescent Psychiatry** 10.1007/s00787-022-02107-2

In the original publication, the first and second name has swapped inadvertently. The correct names are Johanna Timonen, Mika Niemelä, Helinä Hakko, Anni Alakokkare, Sami Räsänen. The original publication has been corrected.

