# Supplementary material for: Social leisure time activities as a mediating link between self-reported psychological symptoms in adolescence and psychiatric morbidity by young adulthood: the Northern Finland 1986 Birth Cohort study
Source: Eur Child Adolesc Psychiatry. 2022 Nov 22;32(12):2569–80. doi: 10.1007/s00787-022-02107-2 (PMC10682069; doi:10.1007/s00787-022-02107-2)
Supplement: Supplementary file 2 — Supplementary file2 (DOCX 16 KB) [file 787_2022_2107_MOESM2_ESM.docx]

Social leisure time activities as a mediating link between self-reported psychological symptoms in adolescence and psychiatric morbidity by young adulthood: the Northern Finland 1986 Birth Cohort study

Social Psychiatry and Psychiatric Epidemiology

Author Affiliation

Timonen Johanna^1^, Niemelä Mika^2,3^, Hakko Helinä^2^, Alakokkare Anni^3^, Räsänen Sami^1,2^

1 Faculty of Medicine, Research Unit of Clinical Neuroscience, Psychiatry, University of Oulu, Finland

2 Department of Psychiatry, Oulu University Hospital, Oulu, Finland

3 Faculty of Medicine, Center for Life Course Health Research, University of Oulu, Oulu, Finland

Corresponding author e-mail: Riika.Timonen@oulu.fi

**Supplementary material,** Appendix 1, Covariates

In the present study sample (n=6,709), 48.1% were males, 78.3% had biological parents living together, 38.1% of their parents had completed at least secondary level education, and 36.9% had at least one parent with a history of doctor-diagnosed psychiatric disorder. Of all study participants, 2,081 (31.0%) belonged to high level of social leisure activity (high SLA) group, 4,422 (65.9%) to middle level of social leisure activity (middle SLA) group, and 206 (3.1%) to low level of social leisure activity (low SLA) group.

The results of comparisons between the three SLA groups showed higher prevalence of males in the high SLA group (53.1%) compared to the low SLA (47.4%) and middle SLA (45.9%) groups (p< 0.001). The proportion of biological parents not living together was significantly lower in the high SLA group (18.3%) compared to 23.2% in both the low- and middle SLA groups (p<0.001). The proportion of parents who had completed at least secondary level education was lowest in the low SLA (38.3%) and middle SLA (33.5%) groups compared to the high SLA (47.3%) group (p<0.001). Parental psychiatric disorders were less common in the high SLA group (34.2%) compared to the low SLA (38.1%) and middle SLA (38.1%) groups (p=0.009).
