# Supplementary material for: Social leisure time activities as a mediating link between self-reported psychological symptoms in adolescence and psychiatric morbidity by young adulthood: the Northern Finland 1986 Birth Cohort study
Source: Eur Child Adolesc Psychiatry. 2022 Nov 22;32(12):2569–80. doi: 10.1007/s00787-022-02107-2 (PMC10682069; doi:10.1007/s00787-022-02107-2)
Supplement: Supplementary file 3 — Supplementary file3 (DOCX 19 KB) [file 787_2022_2107_MOESM3_ESM.docx]

**Supplement table 2** Association of leisure time activity and self-reported mental symptoms using YSR subscales in adolescence with likelihood of psychiatric disorder diagnosed by young adulthood, the 1986 Northern Finland Birth Cohort Study

|  | **F00-F99: Any psychiatric disorder** | **F10-F19: Substance use disorders** | **F20-F29: Psychotic disorders** | **F30-F39: Affective disorders** | **F40-F49: Anxiety** | **F90-F98: Behavioural disorders** |
| --- | --- | --- | --- | --- | --- | --- |
|  | OR (95% CI) | OR (95% CI) | OR (95% CI) | OR (95% CI) | OR (95% CI) | OR (95% CI) |
| Social leisure activity^1^ |  |  |  |  |  |  |
| Low-SLA^2^ | 1.42 (0.97-2.07) | 0.92 (0.39-2.15) | 1.08 (0.38-3.10) | 1.13 (0.69-1.86) | 1.91 *(1.24-2.96)* | 4.30 *(1.84-10.07)* |
| High-SLA^2^ | 0.82 *(0.69-0.97)* | 0.58 *(0.39-0.86)* | 0.91 (0.56-1.49) | 0.77 *(0.61-0.98)* | 0.83 (0.66-1.05) | 1.19 (0.68-2.08) |
| **YSR^3^ subscales:** |  |  |  |  |  |  |
| (1) anxious/depressed | 1.44 *(1.16-1.79)* | 1.01 (0.65-1.58) | 0.65 (0.36-1.17) | 1.50 *(1.14-1.98)* | 1.67 *(1.27-2.21)* | 0.92 (0.46-1.83) |
| (2) withdrawn/depressed | 1.08 (0.89-1.32) | 1.25 (0.84-1.86) | 2.34 *(1.41-3.89)* | 1.30 *(1.01-1.66)* | 1.13 (0.88-1.47) | 0.84 (0.44-1.59) |
| (3) somatic complaints | 1.06 (0.88-1.27) | 1.15 (0.79-1.66) | 1.13 (0.69-1.86) | 1.20 (0.94-1.52) | 1.01 (0.79-1.28) | 1.61 (0.92-2.82) |
| (4) social problems | 1.06 (0.86-1.29) | 0.95 (0.63-1.43) | 1.37 (0.80-2.34) | 1.28 (0.99-1.66) | 0.91 (0.70-1.19) | 0.64 (0.33-1.23) |
| (5) thought problems | 1.07 (0.88-1.31) | 1.23 (0.83-1.81) | 1.57 (0.93-2.65) | 0.97 (0.75-1.26) | 1.01 (0.78-1.31) | 1.71 (0.95-3.09) |
| (6) attention problems | 1.56 *(1.30-1.87)* | 1.75 *(1.21-2.53)* | 1.58 (0.95-2.64) | 1.44 *(1.13-1.82)* | 1.59 *(1.24-2.02)* | 1.75 (0.98-3.11) |
| (7) rule-breaking behavior | 1.15 (0.94-1.40) | 2.05 *(1.41-2.99)* | 1.28 (0.75-2.18) | 1.24 (0.96-1.60) | 0.95 (0.73-1.24) | 1.69 (0.93-3.07) |
| (8) aggressive behavior | 0.97 (0.79-1.20) | 0.93 (0.61-1.40) | 1.30 (0.75-2.27) | 0.98 (0.74-1.29) | 0.96 (0.73-1.27) | 1.26 (0.67-2.36) |
| **Covariates:** |  |  |  |  |  |  |
| Female gender | 1.40 *(1.21-1.64)* | 0.50 *(0.36-0.69)* | 0.57 *(0.36-0.89)* | 1.95 *(1.58-2.40)* | 1.64 *(1.34-2.01)* | 1.18 (0.71-1.93) |
| Biological parents not living together | 1.45 *(1.23-1.72)* | 2.11 *(1.52-2.92)* | 1.03 (0.62-1.70) | 1.46 *(1.17-1.81)* | 1.48 *(1.19-1.85)* | 1.93 *(1.15-3.22)* |
| Parental education secondary school | 1.02 (0.87-1.19) | 0.94 (0.67-1.32) | 1.55 (1.00-2.41) | 1.06 (0.86-1.30) | 1.07 (0.87-1.32) | 0.73 (0.43-1.27) |
| Parental psychiatric disorder | 1.71 *(1.47-1.99)* | 1.81 *(1.31-2.49)* | 2.13 *(1.37-3.32)* | 1.82 *(1.49-2.22)* | 1.74 *(1.42-2.12)* | 1.75 *(1.06-2.88)* |

OR’s calculated with logistic regression with several outcomes. Models adjusted with gender, family structure and parental education when cohort
members were 15–16, and parental psychiatric disorders until the end of 2018, has been built to examine associations of hierarchical hobby and
YSR-subscales at the age of 15–16 with later psychiatric disorders. Participant can have diagnoses from several diagnosis groups. Levels of social leisure
activity are mutually exclusive.
^1^reference category = Middle social leisure activity
^2^SLA social leisure activity
^3^YSR Youth self-report, 82^nd^ percentile cutoff
