# Supplementary figures and images for: Social leisure time activities as a mediating link between self-reported psychological symptoms in adolescence and psychiatric morbidity by young adulthood: the Northern Finland 1986 Birth Cohort study
Source: Eur Child Adolesc Psychiatry. 2022 Nov 22;32(12):2569–80. doi: 10.1007/s00787-022-02107-2 (PMC10682069; doi:10.1007/s00787-022-02107-2)

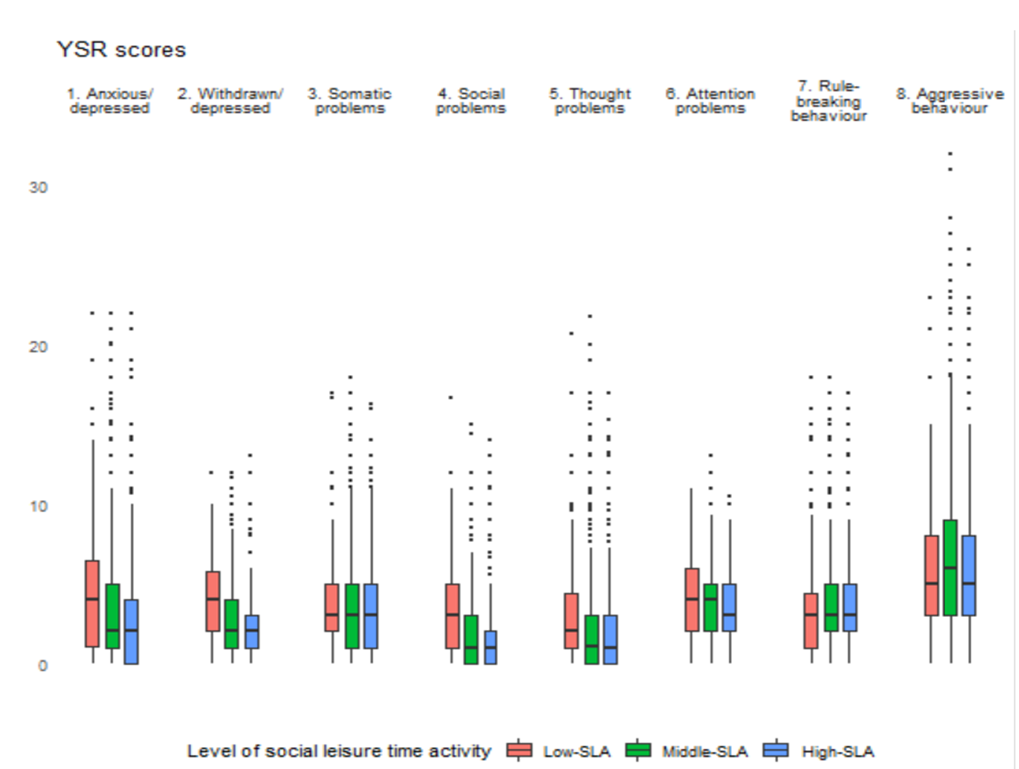

Supplement: Supplementary file 4 — Supplementary file4 (DOCX 145 KB) [file 787_2022_2107_MOESM4_ESM.docx]

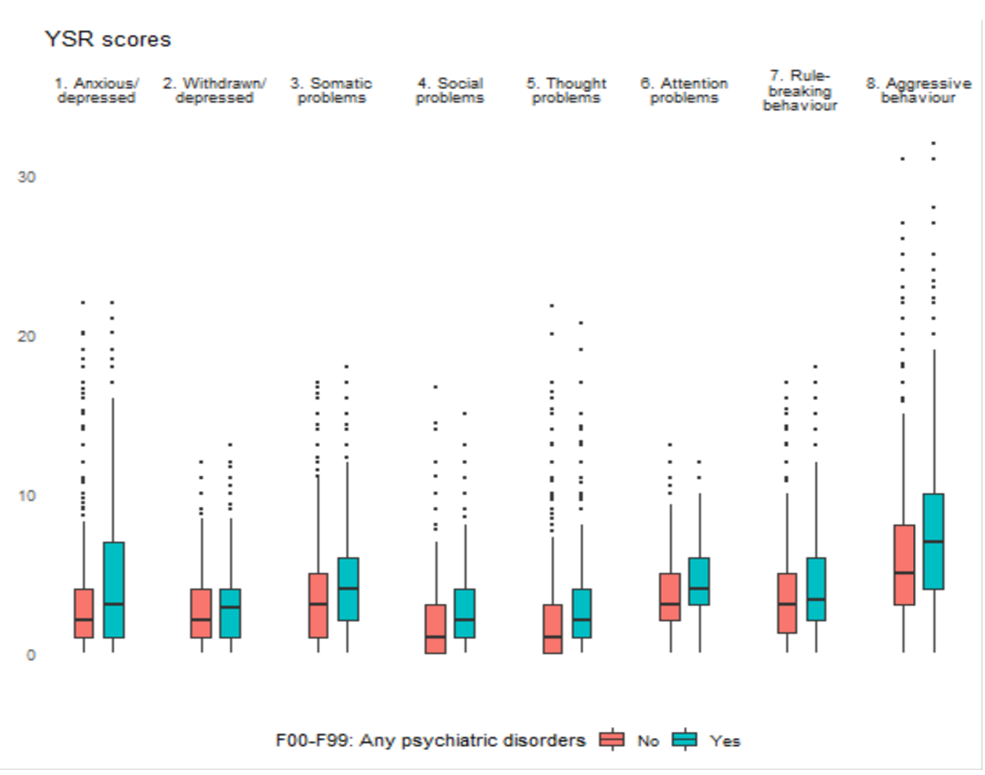

Supplement: Supplementary file 5 — Supplementary file5 (DOCX 130 KB) [file 787_2022_2107_MOESM5_ESM.docx]
